# PLK4 as a potential target to enhance radiosensitivity in triple-negative breast cancer

**DOI:** 10.1186/s13014-024-02410-z

**Published:** 2024-02-16

**Authors:** Sierra Pellizzari, Vasudeva Bhat, Harjot Athwal, David W. Cescon, Alison L. Allan, Armen Parsyan

**Affiliations:** 1 Department of Anatomy and Cell Biology, Western University, N6A 3K7 London, ON Canada; 2 London Regional Cancer Program, London Health Sciences Centre and London Health Sciences, Centre Research Inc, N6A 5W9 London, ON Canada; 3 Princess Margaret Cancer Centre, University Health Network, University of Toronto, M5G 2M9 Toronto, ON Canada; 4 Department of Medical Oncology and Hematology, University of Toronto, M5G 2C1 Toronto, ON Canada; 5 Department of Oncology, Western University, N6A 3K7 London, ON Canada; 6 Department of Surgery, St Joseph’s Health Care and London Health Sciences Centre, Western University, N6A 4V2 London, ON Canada

**Keywords:** Breast Cancer, Centrinone B, CFI-400945, Combination therapy, Radiotherapy, Organoids, PLK4, Triple-negative breast cancer, Centrosome

## Abstract

Radioresistance is one of the barriers to developing more effective therapies against the most aggressive, triple-negative, breast cancer (TNBC) subtype. In our previous studies, we showed that inhibition of Polo-like Kinase 4 (PLK4) by a novel drug, CFI-400945 significantly enhances the anticancer effects of radiotherapy (RT) compared to single treatment alone. Here we further investigate the role of PLK4 in enhancing radiation effects in TNBC and explore mechanisms of PLK4 inhibition and radiation combinatorial antiproliferative effects. To assess cellular proliferation in response to treatments, we used colony formation assays in TNBC cell lines and patient-derived organoids (PDOs). Downregulation of PLK4 expression was achieved using siRNA silencing in TNBC cell lines. Immunofluorescence against centrin was used to assess the alteration of centriole amplification in response to treatments. We observed that inhibition of PLK4 by CFI-400945 or Centrinone B or its downregulation by siRNA, when combined with RT, resulted in a significant increase in antiproliferative effect in TNBC cells lines and PDOs compared to untreated or single-treated cells. Anticancer synergy was observed using a response matrix in PDOs treated with CFI-400945 and RT. We show that the overamplification of centrioles might be involved in the combined antiproliferative action of RT and PLK4 inhibition. Our data suggest that PLK4 is a promising target for enhancing the anticancer effects of RT in TNBC that, at least in part, is modulated by the overamplification of centrioles. These results support further mechanistic and translational studies of anti-PLK4 agents and RT as an anticancer combination treatment strategy.

**Supplementary Information:**

The online version contains supplementary material available at 10.1186/s13014-024-02410-z.

## Introduction

Effective treatment strategies for triple-negative breast cancer (TNBC) are needed, given poor oncological outcomes in this aggressive disease subtype. To improve patient outcomes in TNBC, new therapeutics and multimodality treatments are being investigated. Polo-like Kinase 4 (PLK4), a regulator of centriole duplication [1, 2], was found to be a promising target for drug development in breast cancer [3]. Several PLK4 inhibitors were developed, including an orally available drug, CFI-400945 [3] and a highly specific Centrinone B [4]. CFI-400945 was well-tolerated in a Phase I clinical trial [5] and has entered Phase II studies in metastatic breast cancer (NCT03624543). Inhibition of PLK4 dysregulates centriole duplication and induces genomic instability and aneuploidy in cancer cells, causing antiproliferative effects and cell death [1, 3, 6]. Genomic instability is also a sequela of effects of radiotherapy (RT), a commonly used modality for breast cancer treatment [6, 7]. Ionizing radiation is also known to induce centriole-related aberrations and centrosome overduplication [8, 9]. Thus, the combination of PLK4 inhibition with RT might synergize in dysregulation of centrioles leading to, compared to single-agent treatment, further genomic instability of cancer cells and enhanced antiproliferative effects. In our previous studies, we showed that CFI-400945 and RT indeed can act synergistically in vitro and in vivo to decrease TNBC proliferation [6]. Here, we further investigate if previously observed [6] anticancer synergy of CFI-400945 with RT are specific to PLK4 inhibition since this drug is known for its off-target effects (e.g. inhibition of Aurora Kinase B) [3]. We also investigate various approaches to PLK4 inhibition in various cell models and explore the mechanisms of synergistic antiproliferative effects of CFI-400945 in combination with RT in TNBC. Our data suggest that the combination effect of CFI-400945 with RT in TNBC is driven by inhibition of PLK4, which, together with RT leads to the further overamplification of centrioles compared to single agent treatments. This centriole dysregulation likely leads to further exacerbation of genomic instability and genotoxic stress, which in turn reflects in the observed synergistic effects of combination treatment. Our results support further mechanistic and translational studies of anti-PLK4 agents and RT as a novel multimodality combination treatment strategy in TNBC and potentially other cancers.

## Materials and methods

### Colony and organoid forming assays

Human triple negative breast cancer cell lines, MDA-MB-468 (ExPASy Cellosaurus Research Resource Identifier (RRID): CVCL_0419), MDA-MB-231 (RRID: CVCL_0062) and SUM159 (RRID: CVCL_5423), were used. MDA-MB-468 and MDA-MB-231 cell lines were obtained from Dr. Ann Chambers (London Regional Cancer Program; London, ON). SUM159 cell line was obtained from Asterand Inc. (Detroit, MI, USA). All the cell lines were authenticated via third party testing (IDEXX BioAnalytic, Columbia MO, USA) using short tandem repeat (STR) profiling (using 9 markers) in August 2021. All experiments were performed using mycoplasma-free cell lines. The BPDXO58 and PDO66 organoid lines were provided by the Princess Margaret Cancer Centre Living Biobank (Toronto, ON). Colony and organoid formation assays (CFA) were performed as previously described [10, 11]. Radiation experiments were performed using a Cobalt-60 unit (London Regional Cancer Program, ON) 16–20 h after seeding, media was then replaced and supplemented with the drug (CFI-400945, SelleckChem, PA; Centrinone B, Tocris Bioscience, Bristol, UK) or vehicle control at various concentrations. In pre-treatment combination studies, cells were treated first with RT or the drug 16–20 h after plating, then 4 days later were treated with the drug or RT, respectively. For assessing combination effects in colony formation assays, we used the drug concentration and RT doses closer to IC50/ID50, specific to each cell line. This selection was based on the envisioned translational and biological relevance of these treatments, since IC50/ID50 would represent more clinically relevant concentrations/doses.

### siRNA knockdown studies

Cells were transfected with either scrambled control or *PLK4-*targeting siRNA (GAAGAUAGCAAUUAUGUGU) (Dharmacon, CO) using Lipofectamine RNAiMAX transfection reagent following the manufacturer’s protocol (Invitrogen, MA) and replated in CFA format 16 h after transfection. In parallel, a subset of transfected cells was assessed for *PLK4* knockdown 48 h after transfection using RT-qPCR after RNA isolation using the TRIzol reagent (Invitrogen). Total RNA (1 µg) was reverse transcribed using the Superscript IV VILO master mix (Invitrogen). *PLK4* knockdown was quantified using PLK4 and GAPDH-specific primers (Supp. Table 1) Brilliant III SYBR green qPCR master mix (Agilent Technologies, Inc, CA) on the QuantStudio 3 Real-Time PCR System (Applied Biosystems, MA). The cycle threshold (Ct) values of PLK4 were normalized to GAPDH internal control to calculate ΔC*t* values. The difference in *PLK4* expression between scrambled control and PLK4 knockdown samples were determined by calculating fold change with the 2^− ΔΔC*t*^ method as previously described [12].

### Statistical analysis

All experiments were performed a minimum of three times, unless specified otherwise. Synergy was calculated using Bliss score using SynergyFinder software [13]. Statistical analysis was performed using GraphPad Prism Software V9.0.1 (Dotmatics, CA). Half-maximal inhibitory concentration (IC50, for drugs) and half-maximal inhibitory dose (ID50, for RT) were calculated using dose response curves and a nonlinear regression model (Supp. Figure 1). Statistical analysis of the data from CFA and immunohistochemistry experiments comparing control, single agent and combination treatment effects was performed using Two-Way ANOVA. Statistical significance was defined as *p* ≤ 0.05.

**Fig. 1:**
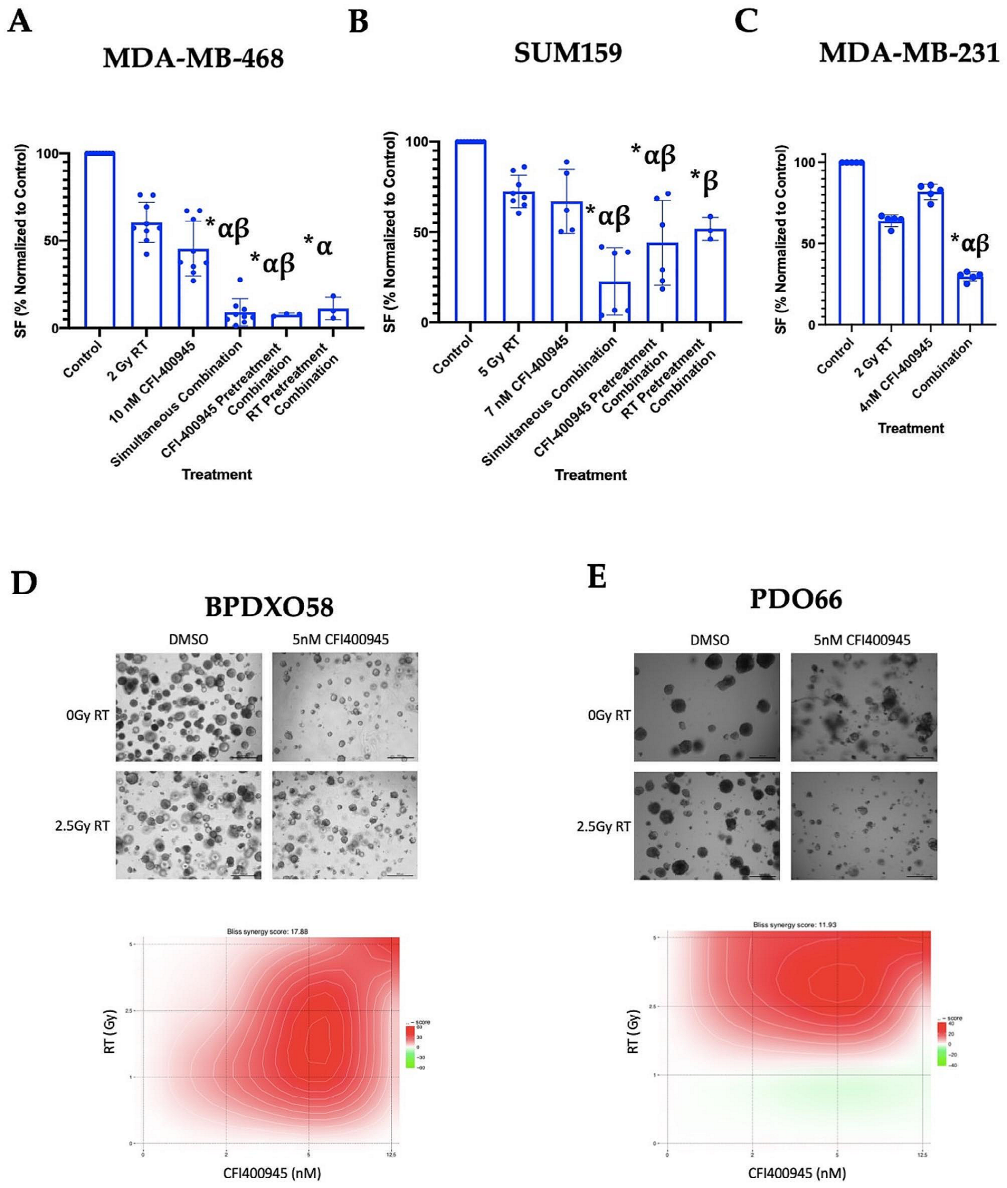
Anticancer effects of CFI-400945 and RT in TNBC cell lines and patient-derived organoids. Combination of CFI-400945 and RT demonstrates significant augmentation of anticancer effect by decreasing colony formation in **(A)** MDA-MB-468, **(B)** SUM159 and **(C)** MDA-MB-231 cells compared to control (*), RT (α) or CFI-400945 (β) only (*p* ≤ 0.05), upon simultaneous or sequential combination treatments. The combination effect was observed and was not significantly altered by the pre-treatment of cells with CFI-400945 or RT compared to simultaneous treatment. Synergy of the CFI-400945 and RT combination treatment was observed in **(D)** BPDXO58 and **(E)** PDO66 at various concentrations of the drug and RT doses using organoid formation assays. Bright-field microscopy images (4× magnification) were taken 14 days following treatment. The number of organoids were counted by 2 independent observers in at least 3 random fields per each well. The counts were normalized to respective controls in each group. Average number of organoids was normalized to that of control (no-RT, no-drug). Bliss synergy scores were calculated with SynergyFinder and are displayed in the heatmap, where intensity of red indicates higher degree of synergy. RT– Radiotherapy, SF– Surviving Fraction. Scale bar = 500 μm

## Results

### CFI-400945 and RT demonstrate combinatorial anticancer effect

In MDA-MB-468, (Fig. 1A) simultaneous combination treatment resulted in a 91.0 ± 5.6% decrease in colony formation compared to control (*p*≤0.05), which translates to an 8.3- and 5.5-fold decrease in colony formation compared to RT or CFI-400945 only treatments respectively. Similar combinatorial effects were observed in other studied cell lines (Fig. 1B**&C**). We evaluated the sequential scheduling of the RT and drug treatments and observed that different sequencing resulted in similar combinatorial effects and did not significantly alter the efficacy of the combination compared to simultaneous administration (Fig. 1A and B). Hence, subsequent experiments were carried out using simultaneous RT and drug administration protocol. In patient-derived models, BPDXO58 (Fig. 1D) and PDO66 (Fig. 1E), we also observed synergy between CFI-400945 and RT at various dose combinations (Bliss synergy scores of 17.88 (BPDXO58) and 11.93 (PDO66)). In BPDXO58, 5 nM of CFI-400945 and 2.5 Gy of RT resulted in a 12.7-fold decrease in organoid formation compared to control treatment, while single agent CFI-400945 and RT at these doses resulted in only a 1.1-fold and 1.2-fold decrease, respectively (Fig. 1D). Similar trends were observed in PDO66 (Fig. 1E).

### Alternative targeting of PLK4 exhibits combination effects with RT

**Fig. 2:**
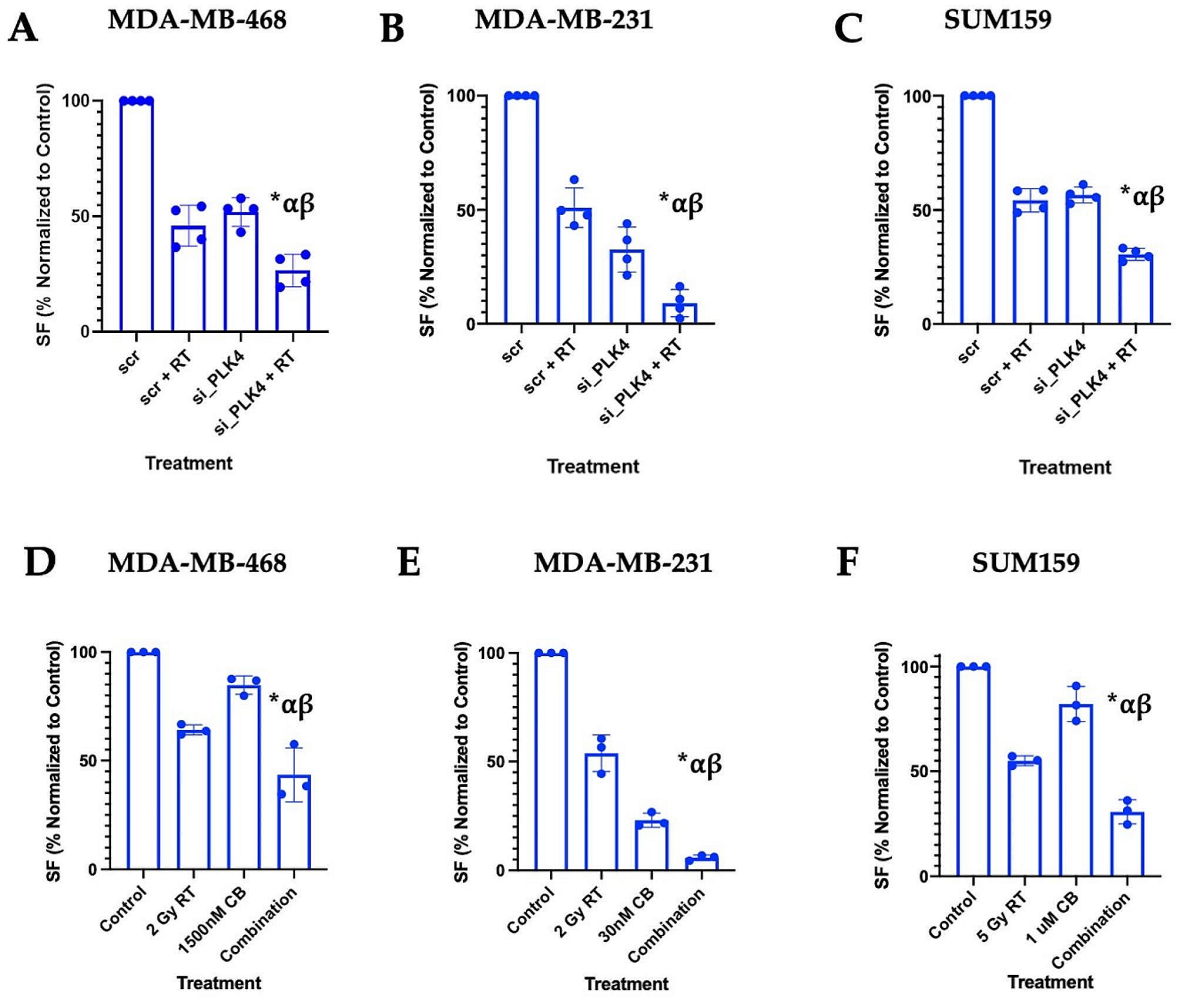
Loss of function or inhibition of PLK4 enhances RT induced anticancer effects. Combination of the PLK4 knockdown and RT demonstrates increased anticancer effects, compared to single-agent treatments in **(A)** MDA-MB-468, **(B)** MDA-MB-231 and **(C)** SUM159 cells transfected with either scrambled control (scr) or PLK4-targeting siRNA (si*PLK4*) and treated with RT (*p* ≤ 0.05 compared to control (*), RT (α) or siPLK4 (β) only). Combination treatment of **(D)** MDA-MB-468, **(E)** MDA-MB-231 and **(F)** SUM159 cells with RT and PLK4 inhibitor Centrinone B results in decreased colony formation compared to control (*), RT (α) or Centrinone B (β) only (*n* = 3, *p* ≤ 0.05). CB– Centrinone B, RT– Radiotherapy, SF– Surviving Fraction

We used PLK4 knockdown with siRNA to investigate if the observed combination effect of CFI-400945 and RT is mediated by PLK4 inhibition and if downregulation of PLK4 expression leads to similar combination effects with RT. In MDA-MB-468, MDA-MB-231 and SUM159 cells, siRNA targeting of *PLK4* resulted in up to 75% reduction of its mRNA and 50–75% average reduction in protein levels (Supp Fig. 2A-C). In MDA-MB-468, combination treatment with RT and PLK4 knockdown resulted in reduction of colony formation by 73.5 ± 7.0% compared to control (*p*≤0.05) (Fig. 2A). Similarly, compared to control, PLK4 silencing together with RT treatment significantly (*p* < 0.05) reduced colony formation by 90.9±6.0% and 69.4±2.6% in MDA-MB-231 and SUM159 cells respectively (Fig. 2B-C).

Next, we investigated the role of PLK4 inhibition in enhancing RT’s antiproliferative effects by another, highly potent PLK4-specific inhibitor, Centrinone B [4]. In MDA-MB-468 cells, the combination of Centrinone B and RT resulted in a 56.6 ± 10.1% decrease in colony formation, whereas 15.2 ± 3.5% and 35.9 ± 1.9% decreases were observed for Centrinone B or RT alone, respectively (*p*≤0.05) (Fig. 2D). Similar effects were observed in other cell lines, where, compared to control, Centrinone B and RT combination treatment significantly (*p* < 0.05) reduced colony formation by 94.2±1.2% in MDA-MB-231 and 69.3±5.8% in SUM159 cells (Fig. 2E **& F**).

### Centriole overduplication may contribute to the combination anticancer effects of CFI-400945 and RT

**Fig. 3:**
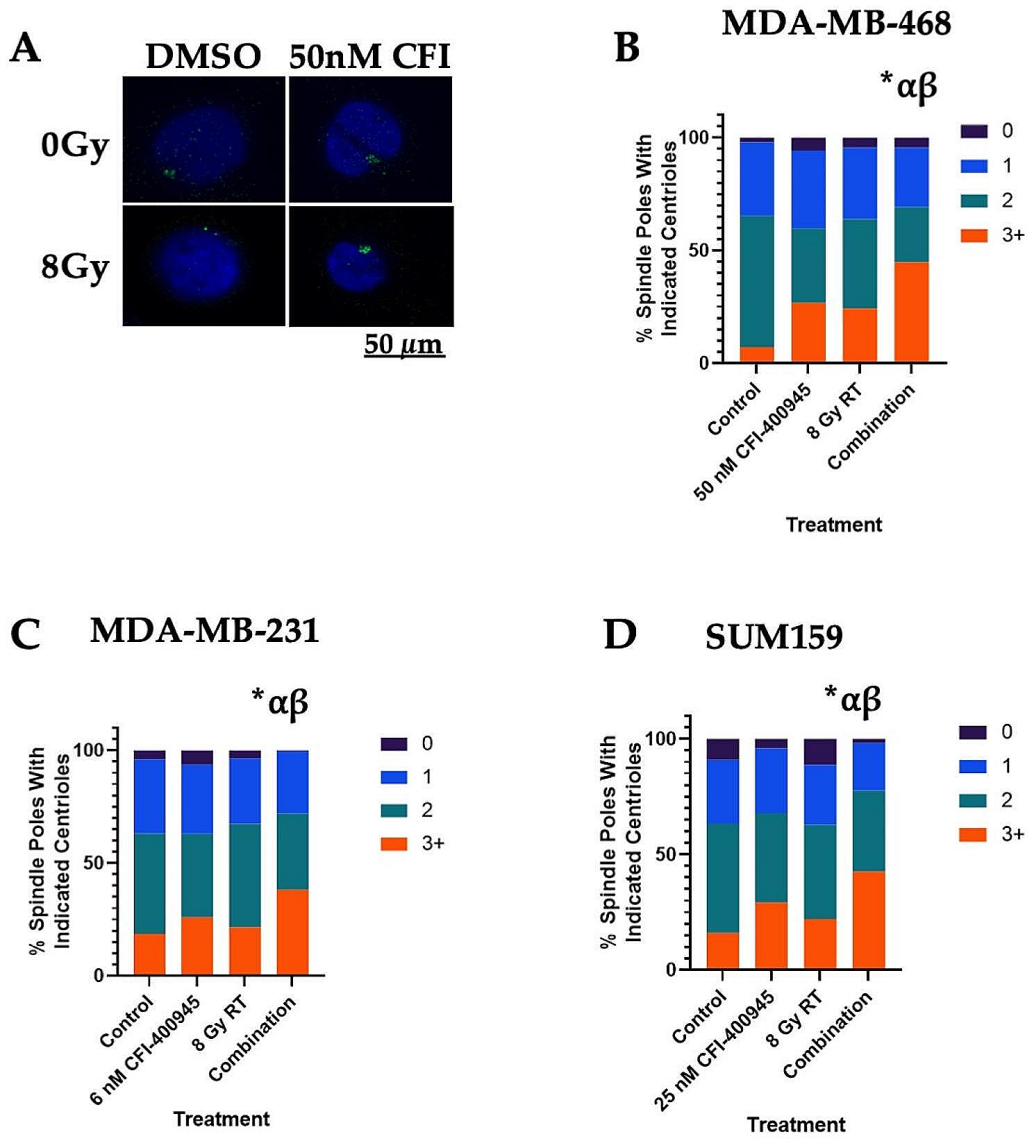
PLK4 inhibition enhances anticancer effects of RT via overamplification of centrioles. **(A)** Representative images depicting nuclear staining by DAPI (blue) and centriole staining by Centrin (green) in MDA-MB-468 cells. Immunocytochemistry for Centrin in **(B)** MDA-MB-468 (*n* = 4), **(C)** MDA-MB-231 (*n* = 3), and **(D)** SUM159 (*n* = 3) cells indicated a significant increase in centriole amplification in combination treatment compared to control (*), RT (α) or CFI-400945 (β) only (*p* ≤ 0.05)

Inhibition of PLK4 by CFI-400945 is known to induce centriole duplication at lower concentrations [3]. Using immunocytochemistry for Centrin, a centriole-associated protein, Mason et al. observed increased numbers of centrioles at spindle poles in MDA-MB-468 cells treated with CFI-400945 [3]. Using a similar experimental approach, we assessed that combination treatment resulted in further overamplification of centrioles (≥3) at spindle poles compared to control in TNBC cell lines (Fig. 3A). In MDA-MB-468 cells, combination treatment resulted in 37.7 ± 4.9% increase in the proportion of cells with centriole overamplification compared to control (*p*≤0.05) (Fig. 3B). Similar effects were observed in other cell lines (Fig. 3C **and D**).

## Discussion

Multimodality combination strategies with chemotherapeutics might improve RT response while providing systemic control in treatment-resistant TNBC [14, 15]. Genomic instability is known to increase cancer cell radiosensitivity [16]. Exploring this vulnerability through combinations of RT and systemic agents is a promising strategy. PLK4 inhibition is an emerging new strategy for cancer, including breast cancer, and has been extensively studied [17, 18]. Various PLK4 inhibitors have been described, such as YLT11 [19] and Centrinone B [20]. However, CFI-400945 is the compound that has been the most extensively studied and has entered clinical trials (NCT03624543) in patients with breast cancer [5]. Mason et al. (2014) [3] characterized CFI-400945, investigating it as a single agent, and provided the foundational concepts regarding this compound. Given its promise as a novel anti-breast cancer agent and orally available drug, our group investigated if the combination of this compound with RT would enhance anticancer effects through further exacerbation of genomic instability [6]. In that study, the first to our knowledge to examine the combination of PLK4 inhibitors with RT, the synergistic effect of CFI-400945 and RT was shown. However, limited models were initially used to test the efficacy of this combination. Moreover, due to potential off-target effects of CFI-400945, it remained to be confirmed that the combination effect was indeed acting directly through PLK4 inhibition. The mechanistic effects of the combination treatment remained unclear. The latter has important translational implications in terms of studying other PLK4 inhibitors with RT to enhance anticancer effects. The aforementioned issues have been addressed in the current study.

Our previous [6] and current data provide strong support that CFI-400945 combined with RT works synergistically to deliver substantially greater anticancer effects than single agent treatments. Synergistic benefits of this combination were confirmed in translationally-relevant PDO models, suggesting that it might be effective in tackling cancer heterogeneity and treatment resistance. In the current manuscript, we provide new information regarding the combination of PLK4 inhibition together with RT.

First, we investigated various sequencing schedules of CFI-400945 with RT and their antiproliferative effects. We did not observe substantial differences in combinatorial effects under various treatment sequencing schedules, likely due to the short periods of culture under various treatment conditions in vitro.

Secondly, we, for the first time, show that specific PLK4 inhibition, at least in part, is responsible for enhanced anticancer effects previously observed upon combination treatment with CFI-400945 and RT. Since CFI-400945 is known to exhibit off-target effects at higher concentrations [4], we explored if PLK4 inhibition is in fact responsible for the antiproliferative synergistic effects with RT using a highly specific PLK4 inhibitor and RNA interference approaches. Reduction of PLK4 expression by siRNA or its inhibition with a highly-selective Centrinone B [4] also enhanced anticancer effects when combined with RT. Although combinatorial effects observed with CFI-400945 were overall stronger compared to siRNA silencing or Centrinone B treatment it is difficult to draw direct comparisons due to experimental differences, such as transient action of siRNA and drug concentrations. Overall, our data suggests that PLK4 is a promising target for enhancing RT effects in TNBC. These findings have important implications for translational research since other PLK4 inhibition strategies are being developed and might soon enter clinical trials [21]. Thus, the knowledge that the PLK4-specific inhibition is a promising strategy for enhancing effects of RT can be further utilized to investigate and develop multimodality treatment approaches in breast and potentially other cancers.

Next, we explored mechanistic aspects of the anticancer effects of PLK4 inhibition with RT. It is worth noting, that while PLK4 is known to play an essential role in centriole and centrosome control, the detailed mechanisms of its action are largely unknown. PLK4 has a known role in cell cycle control by regulating the process of centriole duplication [22]. Hence, inhibition of PLK4 can cause centrosome-related errors, such as centrosome amplification which leads to improper mitotic progression, genomic instability and subsequent cancer cell death [3]. While centriole overduplication is a purported mechanism of the anti-cancer effect of CFI-400945 as a single agent [3], other mechanisms might also be at play during its monotherapy or combination treatment. RT has been shown to induce centrosome amplification in cancer cells via centriole splitting or overamplification [23–25]. However, RT is also known to utilize a multitude of other mechanisms through which it asserts its anticancer effects [15]. Taken together, we aimed to investigate if the combination effect of CFI-400945 inhibition with RT works through centriole overduplication. We, for the first time, show that the combination effect of the drug and RT is, at least in part, promoted by centriole overduplication. The latter is indicative of genomic instability and mitotic catastrophe, which may lead to cancer cell death [24–27].

Our data suggest a model whereby the combined action of PLK4 inhibition and RT leads to increased overamplification of centrioles, which in turn increases genomic instability, compromises the ability of cancer cells to cope with genotoxic stress and results in enhanced anticancer effects. Additional studies might further shed light on the mechanism of action of PLK4 inhibition and RT, serve to identify novel targets for radiosensitization and facilitate translation of this approach to a clinical setting to improve outcomes in patients with TNBC.

Overall, our study shows that PLK4 inhibition together with RT, compared to single agent treatments, further enhances anticancer effects in TNBC. This novel multimodality approach can be potentially utilized in downstaging the primary and axillary lymph node tumors in the neoadjuvant setting, commonly used in TNBC, to improve operability or to convert inoperable tumors to operable ones. This approach might also be proven useful in the metastatic setting (e.g. skin, liver, bones) where radiotherapy effects on the metastatic deposit can be further enhanced by concurrent administration of CFI-400945. Similarly, the combination can be explored in the postoperative, or adjuvant setting to potentially decrease the incidence of locoregional recurrence. The fact that CFI-400945 is orally available makes it an attractive candidate for the multimodal treatment with RT in these settings. Notably, and pending results of Phase II (NCT03624543) trials for CFI-400945 as a single agent, its administration with RT might provide benefits of not only improved loco-regional disease control but also systemic disease control. Due to adverse effects, such as neutropenia, observed upon CFI-400945 treatment in Phase I trials [5], lower doses of the drug could be explored in clinical studies together with RT. Potential benefits of these various management approaches can be further explored in clinical trials with RT and CFI-400945 or other novel PLK4 inhibitors in TNBC. Moreover, our previous [6] and current findings support the rationale for translational and clinical studies of this combination in other breast cancer subtypes and other cancer types.

### Electronic supplementary material

Below is the link to the electronic supplementary material.

Supplementary Material 1. **Supplementary Fig.1.** Dose response curves of single agent treatments in TNBC cell lines Colony formation assays were performed in TNBC cell lines by treating with a range of doses of **(A)** RT, **(B)** CFI-400945 or **(C)** Centrinone B to identify ID50 (RT) or IC50 (drug) values using non-linear regression analysis. The number of colonies counted was normalized to untreated control. SF– Surviving Fraction

Supplementary Material 2. **Supplementary Fig.2.** PLK4 knockdown efficiency in TNBC cells MDA-MB-468 **(A and B)**, MDA-MB-231 **(C and D)** and SUM159 **(E and F)** cells were depleted of PLK4 using siRNA, and the knockdown efficiency was determined by RT-qPCR and Western blot analysis. Reduction of the *PLK4* expression by the siRNA silencing was confirmed by RT-qPCR **(A, C and E)** and immunoblotting **(B, D and F)**. The cycle threshold (Ct) values of PLK4 were normalized to actin internal control (Suppl. Table 1). For immunoblotting, PLK4 band intensity was normalized to the band intensity of actin to calculate normalized band intensity. scr– Scramble control siRNA

Supplementary Material 3

Supplementary Material 4

## Data Availability

Supporting data can be found in the supplementary section of the manuscript.

## Abbreviations

TNBC: Triple-negative breast cancer
PLK4: Polo-like kinase 4
RT: Radiotherapy
PDO: Patient-derived organoids
CFA: Colony and organoid formation assay
Ct: Cycle threshold
IC50: Half-maximal inhibitory concentration
ID50: Half-maximal inhibitory dose
SD: Standard deviation
