# Supplementary figures and images for: PLK4 as a potential target to enhance radiosensitivity in triple-negative breast cancer
Source: Radiat Oncol. 2024 Feb 16;19:24. doi: 10.1186/s13014-024-02410-z (PMC10873955; doi:10.1186/s13014-024-02410-z)

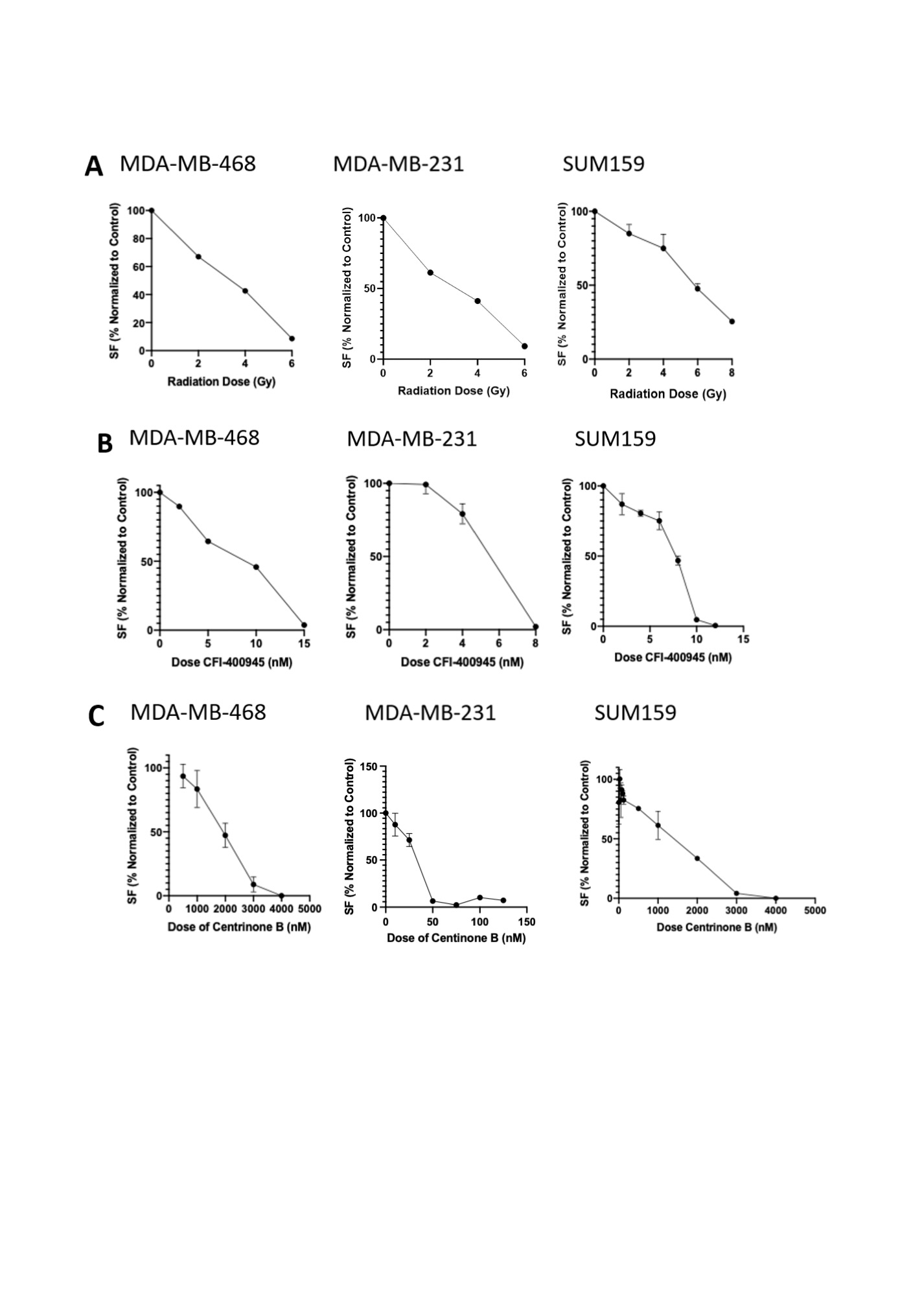

Supplement: Supplementary file 1 — Supplementary Material 1. Supplementary Fig.1. Dose response curves of single agent treatments in TNBC cell lines Colony formation assays were performed in TNBC cell lines by treating with a range of doses of (A) RT, (B) CFI-400945 or (C) Centrinone B to identify ID50 (RT) or IC50 (drug) values using non-linear regression analysis. The number of colonies counted was normalized to untreated control. SF– Surviving Fraction [file 13014_2024_2410_MOESM1_ESM.jpeg]

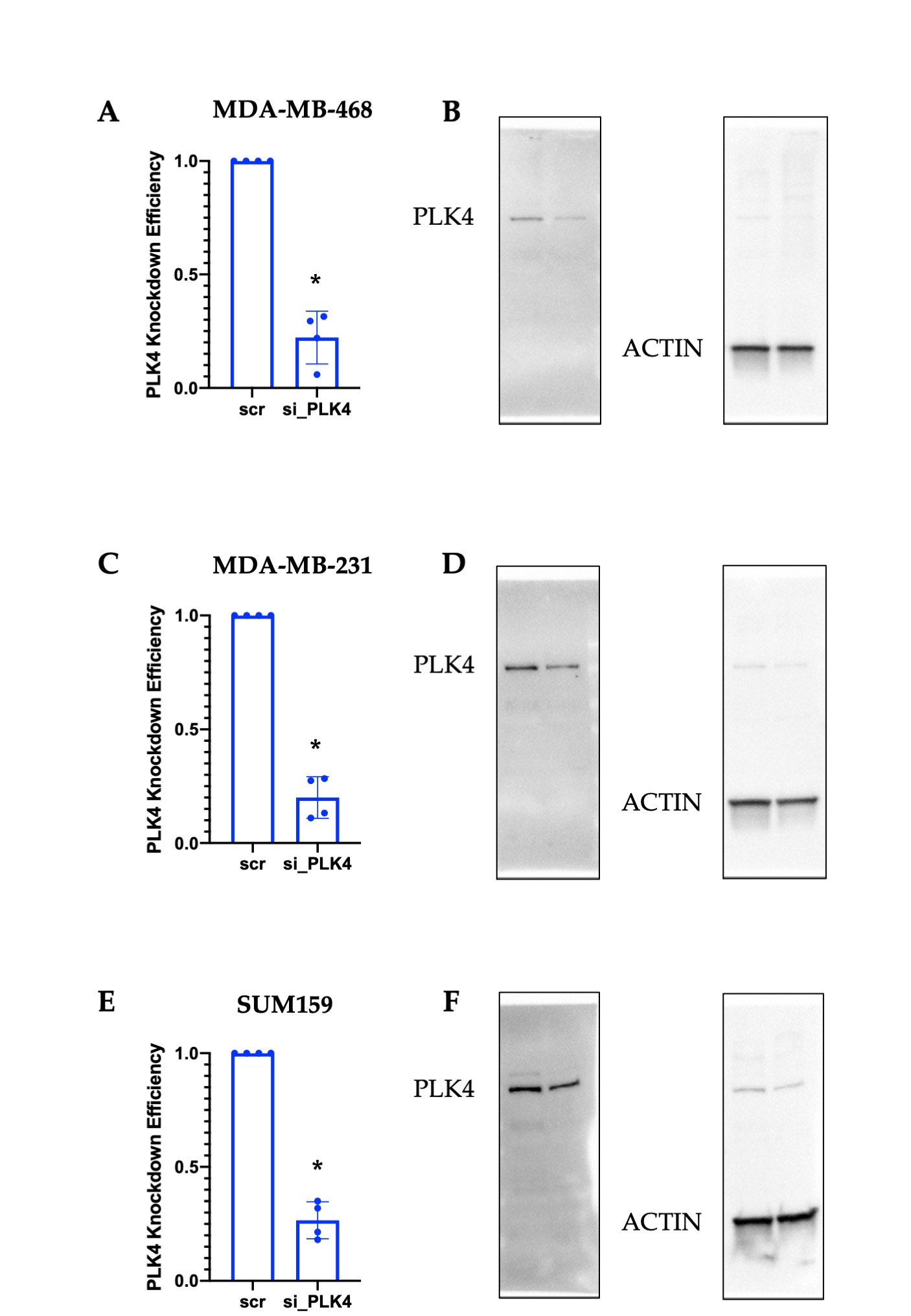

Supplement: Supplementary file 2 — Supplementary Material 2. Supplementary Fig.2. PLK4 knockdown efficiency in TNBC cells MDA-MB-468 (A and B), MDA-MB-231 (C and D) and SUM159 (E and F) cells were depleted of PLK4 using siRNA, and the knockdown efficiency was determined by RT-qPCR and Western blot analysis. Reduction of the PLK4 expression by the siRNA silencing was confirmed by RT-qPCR (A, C and E) and immunoblotting (B, D and F). The cycle threshold (Ct) values of PLK4 were normalized to actin internal control (Suppl. Table 1). For immunoblotting, PLK4 band intensity was normalized to the band intensity of actin to calculate normalized band intensity. scr– Scramble control siRNA [file 13014_2024_2410_MOESM2_ESM.jpeg]
