# Supplementary material for: PLK4 as a potential target to enhance radiosensitivity in triple-negative breast cancer
Source: Radiat Oncol. 2024 Feb 16;19:24. doi: 10.1186/s13014-024-02410-z (PMC10873955; doi:10.1186/s13014-024-02410-z)
Supplement: Supplementary file 4 — Supplementary Material 4 [file 13014_2024_2410_MOESM4_ESM.docx]

**Supplementary Information**

Methods

*Immunoblotting*

Breast cancer cells, transfected as described above, were lysed using 1X RIPA buffer and the resulting total protein was quantified using Lowry assay. Approximately 30µg of protein was subjected to sodium dodecyl sulfate polyacrylamide gel electrophoresis and transferred onto a polyvinylidene difluoride membrane. Membrane was blocked using 5% bovine serum albumin for 1 hour at room temperature in tris-buffered saline + 0.1% tween-20. PLK4 and Actin expression was detected using mouse anti-human PLK4 antibody (Cat. No. MABC544, Clone ID: 6H5, Millipore Sigma, Darmstadt, Germany) and rabbit polyclonal anti-Actin antibody (Cat. No. A2066, Millipore Sigma, Darmstadt, Germany). Protein expression was visualized using Amersham ECL prime detection reagent (GE Healthcare, WI, USA) as per manufacturer's instruction.

*Immunofluorescence*

Immunocytochemistry was performed as previously described(1). Cells seeded on glass coverslips were treated with RT 16-20 hours later, followed by immediate media change containing CFI-400945 or vehicle control. An anti-Centrin, clone 20H5 (Sigma-Aldrich, MI) antibody was used with 1:1000 dilution. Alexafluor 488 anti-mouse secondary antibody (Invitrogen) was used in 1:500 dilution. Coverslips were mounted using Vectashield with DAPI. Images were taken using Nikon Eclipse Ti2 Confocal microscope.

**Reference**

1. Mason JM, Lin DC, Wei X, Che Y, Yao Y, Kiarash R, et al. Functional characterization of CFI-400945, a Polo-like kinase 4 inhibitor, as a potential anticancer agent. Cancer Cell. 2014;26(2):163-76.
